# Saccadic modulation of stimulus processing in primary visual cortex

**DOI:** 10.1038/ncomms9110

**Published:** 2015-09-15

**Authors:** James M. McFarland, Adrian G. Bondy, Richard C. Saunders, Bruce G. Cumming, Daniel A. Butts

**Affiliations:** 1 Department of Biology and Program in Neuroscience and Cognitive Science, University of Maryland, College Park, Maryland 20742, USA; 2 Laboratory of Sensorimotor Research, National Eye Institute, National Institutes of Health, Bethesda, Maryland 20892, USA; 3 Brown-NIH Neuroscience Graduate Partnership Program, Brown University, Providence, Rhode Island 02912, USA; 4 Laboratory of Neuropsychology, National Institute of Mental Health, National Institutes of Health, Bethesda, Maryland 20892, USA

## Abstract

Saccadic eye movements play a central role in primate vision. Yet, relatively little is known about their effects on the neural processing of visual inputs. Here we examine this question in primary visual cortex (V1) using receptive-field-based models, combined with an experimental design that leaves the retinal stimulus unaffected by saccades. This approach allows us to analyse V1 stimulus processing during saccades with unprecedented detail, revealing robust perisaccadic modulation. In particular, saccades produce biphasic firing rate changes that are composed of divisive gain suppression followed by an additive rate increase. Microsaccades produce similar, though smaller, modulations. We furthermore demonstrate that this modulation is likely inherited from the LGN, and is driven largely by extra-retinal signals. These results establish a foundation for integrating saccades into existing models of visual cortical stimulus processing, and highlight the importance of studying visual neuron function in the context of eye movements.

## Abstract (web summary)

Primates acquire visual information through rapid saccadic eye movements, although little is known about their effects on neural processing of visual inputs. Here the authors demonstrate that saccades produce modulations of visual cortical processing that likely originate in the thalamus.

## Graphical abstract

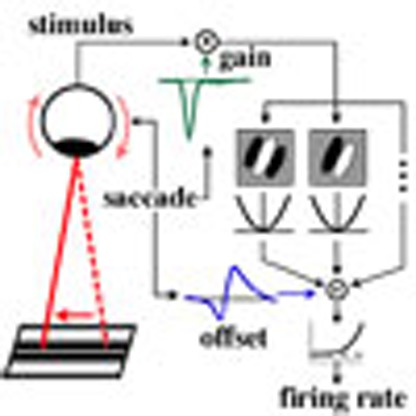

A key component of primate vision is the active sampling of visual inputs by rapid eye movements known as saccades. Saccades cause discontinuous jumps in the visual image on the retina, yet the visual system assimilates this constantly shifting visual input into a seamless percept, suggesting that specific mechanisms exist to integrate visual stimuli with information about eye movements^1^. Indeed, extra-retinal signals associated with saccades have been identified throughout much of the visual processing hierarchy^2^,^3^,^4^,^5^,^6^,^7^,^8^,^9^,^10^, beginning with the lateral geniculate nucleus (LGN)^11^,^12^,^13^. Relatively little is known, however, about whether and how such signals shape visual cortical processing, particularly in early visual areas.

The detailed functional models that have been developed for the primary visual cortex (V1)^14^ make it an ideal system for studying the effects of saccades on stimulus processing. Such studies are complicated, however, by the fact that rapid changes in the retinal stimulus created by saccades strongly modulate the activity of V1 neurons^15^,^16^,^17^, and thus stimulus-driven effects occur simultaneously with any extra-retinal signals. As a result, most studies of saccade modulation in V1 have focused on comparing neural responses between two conditions: (1) when a ‘preferred’ stimulus (typically a bar or grating) is introduced by a saccade; and (2) when the same stimulus is flashed in (or swept over) the receptive field (RF) during fixation. These studies have generally found little difference between the two conditions^5^,^18^,^19^,^20^,^21^,^22^, leading to the belief that modulatory signals associated with saccades are unlikely to play a significant role in V1 activity during normal vision.

There are several important limitations of these previous studies, however. First, saccades introduce uncontrolled variability in the precise spatiotemporal stimulus on the retina, making direct comparisons with stimuli presented during fixation difficult to interpret. Second, previous studies have only looked at the effects of saccades on average firing rates, and thus did not attempt to distinguish between different functional forms of saccade modulation (for example, ‘additive’ versus ‘multiplicative’ interactions), which could have very different implications for perisaccadic encoding of stimulus information. In fact, V1 neurons typically show complex nonlinear stimulus processing that can be described in terms of multiple ‘subunit inputs’^23^, each of which may in principle be modulated in different ways by saccades. Therefore, addressing the more general question of saccadic modulation of stimulus processing requires measuring how saccades alter V1 responses to a broad range of stimuli, and ultimately integrating such perisaccadic modulation into the detailed functional models that have been developed to describe V1 stimulus processing.

Here we address both of these limitations using a functional modelling approach, combined with an experimental design that allows precise control of the retinal stimulus independent of saccades. Two key innovations allow us to disentangle stimulus-driven and saccade-driven effects. First, we used one-dimensional noise stimuli comprised of long bars, while the animals made cued saccades parallel to the bar stimuli^24^,^25^. Thus, during a single video frame, the saccades produced minimal changes to the retinal stimulus. Second, the stimulus consisted of independent noise patterns on each video frame, so that across frames a neuron’s RF was presented with a sequence of independent noise images, regardless of saccades^26^. This stimulus also allowed for us to use recently developed statistical techniques to estimate nonlinear stimulus processing in V1. These models reveal a much richer interaction between saccades and visual stimuli than possible with previous studies.

We found that saccades produced a robust biphasic modulation of average V1 firing rates in response to visual stimuli, with an initial decrease followed by an increase. Using functional models, we show that this biphasic modulation is composed of two distinct effects on stimulus processing: divisive gain suppression, and a stimulus-independent (additive) increase in firing rates. These changes resulted in a sharply reduced stimulus selectivity that lasted for over 100 ms.

We also used this approach to analyse how V1 processing is affected by microsaccades, which are small involuntary saccades produced during fixation^27^,^28^. The effects of microsaccades on V1 stimulus processing remain greatly debated^28^,^29^,^30^, likely because of similar difficulties disentangling such effects from the impact of microsaccades on the retinal stimulus. Leveraging our use of a rapidly flashed noise stimulus that minimizes microsaccade-induced retinal image motion^26^, we found that microsaccades produced similar firing rate modulation compared with saccades, although with weaker reductions of stimulus selectivity.

Furthermore, we present several different lines of evidence that this saccade modulation arises via the LGN. First, the timing of perisaccadic firing rate modulation across neurons and cortical layers is closely related to the timing of the neurons’ stimulus processing. Second, detailed analyses of the functional form of perisaccadic gain suppression demonstrate that it must be inherited from the neurons’ stimulus-driven inputs, rather than from any direct modulatory signals. By manipulating saccade-driven visual stimuli in the far surround, we also demonstrate that the bulk of this saccade modulation was produced by extra-retinal signals.

In total, our results demonstrate clear modulation of V1 stimulus processing by saccades and microsaccades, and illustrate how such effects can be incorporated into existing functional models to provide more general descriptions of V1 processing in the context of eye movements.

## Results

### Saccades produce biphasic modulation of V1 firing rates

**Figure 1:**
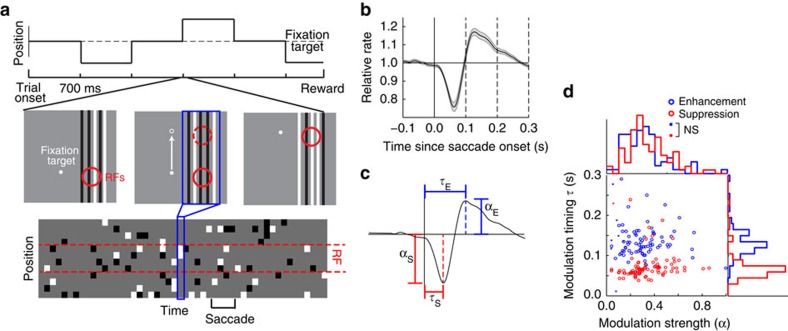
Saccades produce biphasic modulation of V1 neuron firing rates. (**a**) Schematic of the behavioural task and stimulus. Top: the animal was required to maintain fixation on a target that made periodic jumps every 700 ms. Middle: random bar patterns (‘one-dimensional (1D) ternary noise’ updated every 10 ms) were displayed, covering the recorded neurons’ RFs, while the animal made ‘guided saccades’ to maintain fixation on a visual target. Bottom: because the fixation target moved parallel to the random bar stimuli, the sequence of 1D noise patterns in the neurons’ RFs (region highlighted by dashed red lines) were not affected by accurately executed saccades (timing of an example saccade indicated below). (**b**) Saccade-triggered average SU firing rates (normalized by each neuron’s mean rate) showing biphasic modulation (*n*=84). Here, and in all subsequent figures, shaded regions show the interval mean±s.e.m. (**c**) Schematic showing the definitions of suppression and enhancement magnitudes (*α*_S_ and *α*_E_, respectively), as well as their timing (*τ*_S_ and *τ*_E_). (**d**) For neurons with significant modulation (circles; suppression: *n*=83 out of 84; enhancement: *n*=77 out of 84; see Methods), the strengths of perisaccadic suppression and enhancement were variable, but had similar magnitudes overall (suppression: 0.32, 0.24–0.44; enhancement: 0.29, 0.19–0.38; median, interquartile range). The timing of peak saccadic suppression was highly conserved across neurons (64, 56–72 ms), while the timing of peak enhancement was more variable (127, 112–154 ms). Small dots indicate non-significant peaks.

We recorded the activity of macaque V1 neurons using multielectrode arrays, while subjects made periodic ‘guided’ saccades (every 700 ms) to maintain fixation on a small target (Fig. 1a). During this time, a ‘one-dimensional’ ternary noise stimulus was displayed, consisting of uncorrelated random patterns of long narrow bars (whose orientation was matched to the recorded neurons’ preferred orientation) updated at 100 Hz. Two features of this design combine to minimize any effect of eye movements on the retinal stimulus. First, using a noise stimulus that is uniform and uncorrelated in space and time, we ensure that the stimulus in a neuron’s RF is an independent, identically distributed noise pattern on every video frame, regardless of any inter-frame changes in eye position. Second, because the fixation target shifted along the long axis of the bar stimuli^24^,^25^, saccades produced minimal displacement of the bars during the phosphor persistence of any one frame (Fig. 1a). This experiment thus allowed us to directly gauge the effects of saccades on visual responses, independent of the changes that they typically induce in the stimulus itself.

We first examined the effects of these guided saccades on the average firing rates of V1 neurons. Across the population (*n*=84), saccades produced a clear biphasic modulation of average rates, with a rapid initial suppression followed by enhancement (Fig. 1b). The overall biphasic form of the modulation, as well as its timing, was consistent across neurons (Fig. 1c,d), with the exception of a small fraction of neurons (8%) that showed roughly opposite-sign modulation (Supplementary Fig. 1). Furthermore, the strength of both suppression and enhancement varied substantially across neurons, with some neurons showing firing rate increases and/or decreases of more than 50%, though typical modulation was about 30%.

Although our experiment was designed such that accurately executed saccades produced no change to the stimulus in the neurons’ RFs, we verified that any stimulus motion due to inaccuracies in the animals’ saccades did not contribute to the observed perisaccadic modulation (Supplementary Fig. 2). First, we computed the average power spectrum of the stimulus on the retina during saccades (taking into account the measured phosphor response of our displays) and verified that saccades introduced minimal changes to relevant statistics of the stimulus ensemble. Second, we compared the firing rate modulation produced by the most accurate and least accurate saccades (using a median split), and found that they produced nearly identical perisaccadic modulation.

These measurements therefore demonstrate that saccades produced substantial changes in the average responses of V1 neurons, independent of saccade-related changes to the stimulus in the neurons’ RF.

### Monophasic gain suppression and firing rate increase

The above results show the effects of saccades on average firing rates in V1, but these changes in firing rate may not indicate anything about how well visual stimuli are represented around the time of saccades. For instance, perisaccadic changes in firing rate could be independent of the stimulus (additive), or they could arise due to changes in response gain (multiplicative). Critically, our experimental design allows us to address this question by ensuring that we can reconstruct the precise spatiotemporal stimulus on the retina^31^, and thus make detailed measurements of V1 neuron stimulus processing during the guided saccade task.

**Figure 2:**
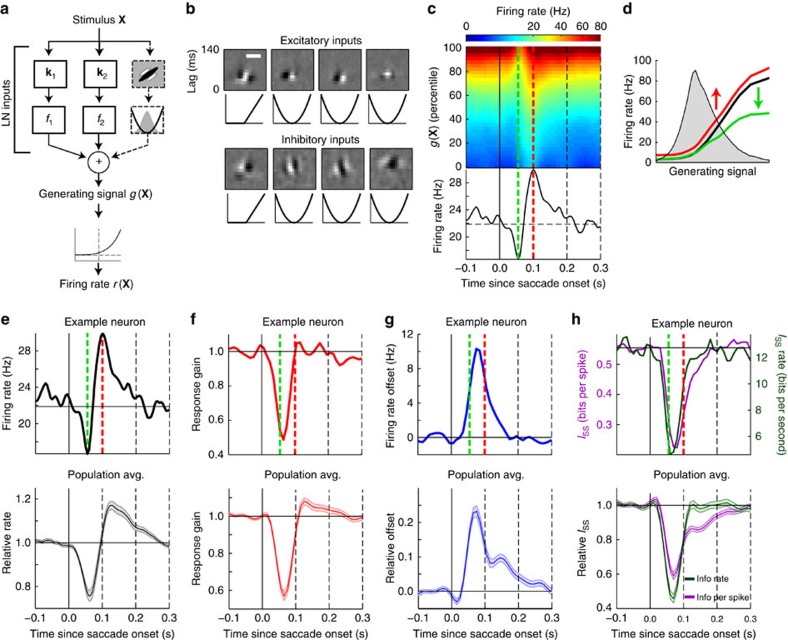
Saccades produce a monophasic suppression of gain and stimulus information. (**a**) Schematic of the nonlinear LNLN cascade stimulus processing models. The summed input of a set of LN subunits gives the ‘generating signal’ *g*(*t*), which is transformed into a firing rate by the spiking nonlinearity. (**b**) Example SU stimulus processing model with 4 excitatory (top) and 4 suppressive (bottom) filters. The ‘upstream nonlinearity’ associated with each filter is shown below. Scale bar is 0.2°. (**c**). Firing rate (colour) as a joint function of the generating signal and time since saccade onset for the example neuron in **b**. Average firing rate at each time relative to saccade onset is shown below. (**d**) Vertical slices from the joint response function show that at the time of maximal saccadic suppression (dashed green line in **c**) the response gain is greatly suppressed (green trace), whereas at the time of maximal saccadic enhancement (dashed red line in **c**), the neuron’s firing rate increases in a largely stimulus-independent manner (red trace). Shaded grey area indicates the distribution of *g*. (**e**) For the example neuron (top), as well as across the population (bottom; *n*=84), saccades produced biphasic firing rate modulation. Such firing rate modulation is decomposed into a multiplicative gain suppression (**f**) and an additive increase in firing rate ‘offset’ (**g**). (**h**) As a result, the single-spike information (*I*_SS_; magenta) showed a large monophasic suppression following saccades (top: example neuron; bottom: population avg.). Information rates (green), given by multiplying *I*_SS_ by saccade-triggered average firing rates, showed similar monophasic suppression.

We first characterized each neuron’s stimulus selectivity using a nonlinear analogue of reverse correlation analysis, whereby its spike probability is given in terms of a set of nonlinear processing ‘subunits’ (an ‘LNLN’ cascade model; Fig. 2a)^32^,^33^. Specifically, we used modified quadratic models (Fig. 2b), which facilitate robust and efficient characterization of the range of V1 stimulus processing from simple cell to complex-cell type responses (Methods).

After inferring the set of spatiotemporal filters for each neuron, we used nonparametric methods to estimate how the summed output of these stimulus processing subunits, the ‘generating signal’ *g*(*t*), was transformed into a firing rate at each time lag *τ* relative to saccade onset. The corresponding two-dimensional response function *r*(*g*,*τ*) (Fig. 2c) reveals that post-saccadic suppression was strongest for ‘preferred stimuli’ (large values of *g*), whereas subsequent firing rate enhancement was largely independent of the stimulus (Fig. 2d). This suggests that saccadic suppression is in the form of a gain change, whereas saccadic enhancement is consistent with a change in firing rate ‘offset’.

To directly assess the extent to which perisaccadic modulation was ‘gain-like’ versus ‘offset-like’, we estimated the perisaccadic response function in relation to the overall response *r*_0_(*g*) (independent of saccades) as:




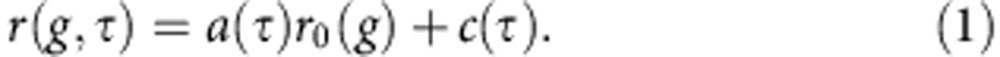




The coefficients *a*(*τ*) and *c*(*τ*) directly capture the multiplicative (‘gain’) and additive (‘offset’) components of saccade modulation, respectively. This showed that the neurons’ biphasic firing rate modulation (Fig. 2e) consists of a monophasic suppression of response gain (Fig. 2f) and a (slightly delayed) monophasic increase in response ‘offset’ (Fig. 2g). In fact, we found that these perisaccadic changes in stimulus processing could be well described by simple models with temporal ‘kernels’ capturing the additive and multiplicative effects of saccades (Supplementary Fig. 3). Incorporating these effects directly into our stimulus processing models also establishes a powerful framework for exploring more detailed aspects of how saccades modulate V1 stimulus processing, as described further below.

### Independent modulation of firing rate and stimulus selectivity

The gain and offset changes described above have different implications for the neurons’ stimulus selectivity, as determined by the distribution of stimuli that trigger spikes. Namely, changes in gain do not affect stimulus selectivity because they scale the firing rate uniformly in response to all stimuli. In contrast, changes in offset imply that additional spikes are not driven by the stimulus, and thus reduce stimulus selectivity by ‘flattening’ the distribution of stimuli signalled by a spike. To quantify perisaccadic changes in stimulus selectivity, we use the single-spike information (*I*_SS_)^34^, which measures the entropy of the spike-triggered stimulus distribution relative to that of the overall stimulus distribution. Note that while we use *I*_SS_ primarily as a measure of stimulus selectivity, it also provides a convenient approximation of the mutual information between spikes and the stimulus, based only on the mean spike rates in response to each stimulus^34^.

Consistent with the observed increase in firing rate offset, saccades produced a monophasic decrease in stimulus selectivity that lasted throughout the period of firing rate modulation (Fig. 2h). While *I*_SS_ measures the stimulus selectivity of individual spikes (and is thus invariant to multiplicative rescaling of the firing rate), we also calculated a proxy for the rate of information transmission by multiplying *I*_SS_ by the average firing rate at each time lag *τ*. Despite the biphasic modulation of average firing rates, we found that saccades also produced monophasic reductions in this measure of the information rate (Fig. 2h).

**Figure 3:**
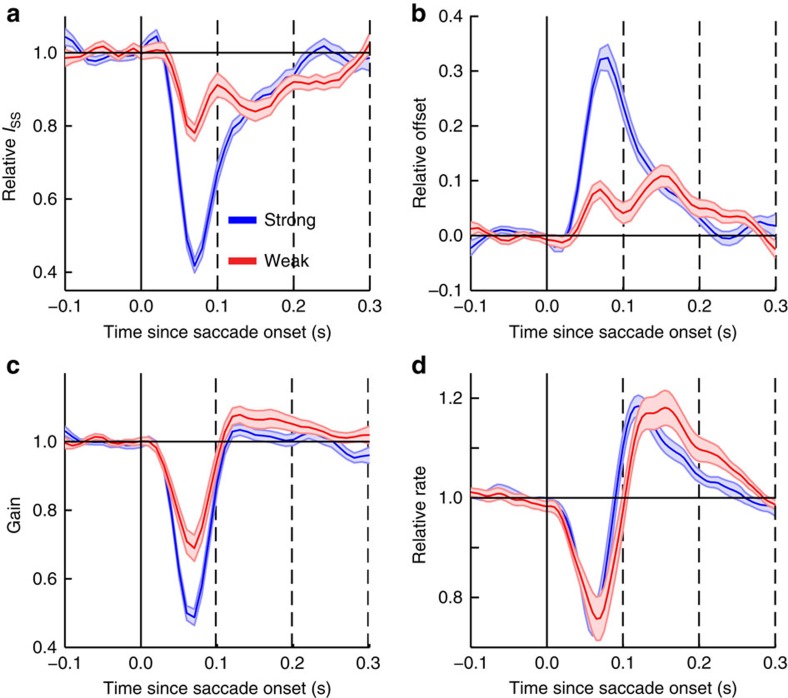
Differential modulation of firing rate and stimulus selectivity. (**a**) Perisaccadic *I*_SS_, computed separately for those neurons showing strong *I*_SS_ suppression (blue; *n*=42) versus weak *I*_SS_ suppression (red; *n*=42), based on a median split. (**b**) Neurons showing strong *I*_SS_ suppression had much larger increases in firing rate offset, as expected. (**c**) Neurons with larger reductions in *I*_SS_ also showed stronger gain suppression. (**d**) Despite large differences in the magnitude of perisaccadic reductions in stimulus selectivity, saccades produced similar firing rate modulation for the two groups of neurons.

These results highlight the fact that firing rate changes are not necessarily predictive of changes in stimulus selectivity. To further illustrate this point, we divided neurons into two groups based on the strength of *I*_SS_ suppression (Fig. 3a). As expected, those neurons showing strong reductions in stimulus selectivity had much larger increases in firing rate offset (Fig. 3b). While *I*_SS_ is in principle independent of gain, neurons that had stronger *I*_SS_ suppression also had stronger gain suppression (Fig. 3c). As a result, these two groups of neurons showed very similar firing rate modulation (Fig. 3d), despite large differences in *I*_SS_ modulation. In fact, the magnitude of *I*_SS_ suppression was slightly negatively correlated with the magnitude of firing rate suppression (Spearman’s rank correlation *ρ*: −0.25, *P*=0.022) and enhancement (*ρ*: −0.24; *P*=0.030). The largely independent perisaccadic changes in firing rate and stimulus selectivity thus emphasize the importance of characterizing the effects of saccades on stimulus processing, in addition to average rates.

### Microsaccades produce similar, but weaker, modulation

A growing body of evidence suggests that involuntary microsaccades are generated by similar circuitry as saccades^35^, and serve analogous functional roles^28^,^36^,^37^. Thus, we next sought to test whether microsaccades, which animals made continually during our experiments (average rate 1.1 Hz), produced similar modulation of V1 stimulus processing. Because microsaccades are involuntary, they were not generally made parallel to the random bar stimulus. However, the short duration of each stimulus frame again minimized any translational stimulus motion during the microsaccades^26^ (Supplementary Fig. 2d). We also utilized a recently developed algorithm to accurately track the component of fixational eye movements orthogonal to the bar stimuli^31^, which was important for an unbiased analysis of their effects on stimulus processing (Supplementary Fig. 4).

**Figure 4:**
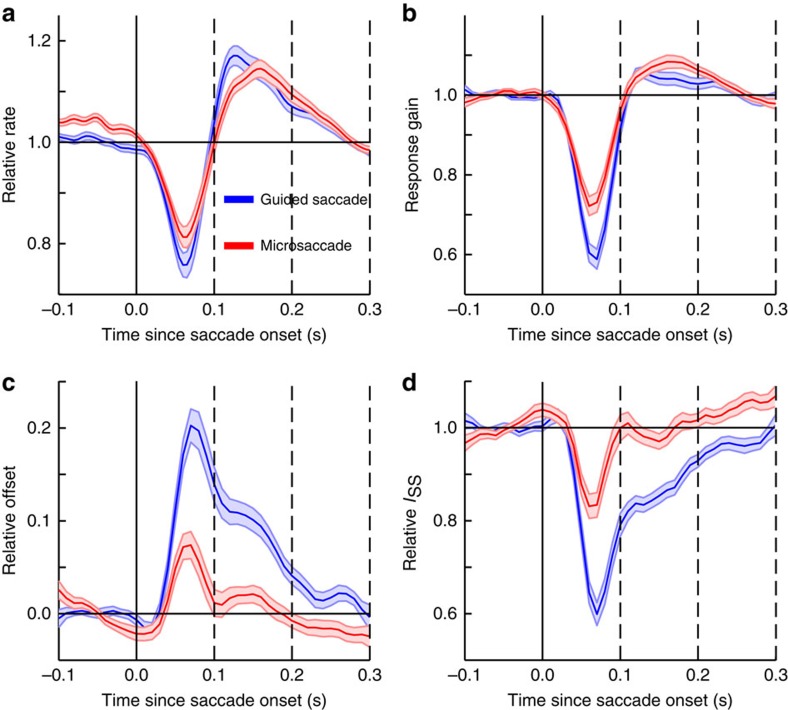
Microsaccades produce similar firing rate modulation, but weaker gain suppression. (**a**) Saccades (blue) and microsaccades (red) produced similar biphasic firing rate modulation, with slightly weaker suppression (median relative suppression magnitude: 0.81-fold; *n*=84) and enhancement (0.77-fold) following microsaccades. (**b**) Suppression of response gains following microsaccades was qualitatively similar to, but weaker than, suppression following saccades. (**c**) The increase in firing rate ‘offset’ following microsaccades was substantially weaker than that following saccades. (**d**) Stimulus information showed monophasic suppression following microsaccades, but the suppression was substantially weaker (0.54-fold) and shorter-lived compared with saccades.

We found that microsaccade- and saccade-triggered average firing rates were remarkably similar (Fig. 4a), with microsaccades producing only slightly weaker suppression (median 0.81-fold) and enhancement (0.77-fold) compared with guided saccades. Although the timing of microsaccadic suppression was similar to saccadic suppression, peak firing rate enhancement following microsaccades was significantly delayed compared with saccades (median difference 14 ms, *P*=1.6 × 10^−4^).

Microsaccades also produced changes in V1 stimulus processing whose features were qualitatively similar to those of saccades, including gain suppression (Fig. 4b) and an increase in response offset (Fig. 4c). However, microsaccade-induced changes, particularly the increased response offset, were substantially weaker than for saccades. As a result, microsaccades produced a suppression of stimulus selectivity that was substantially reduced in magnitude and duration compared with saccades (Fig. 4d). Again, this illustrates the importance of characterizing the effects of eye movements on stimulus processing. The comparison of mean rates in Fig. 4a gives the impression that microsaccades and saccades have very similar effects. Figure 4b–d shows that in fact there is a substantial difference in their effect on the information available about the visual stimulus.

### Timing and laminar profile suggest a geniculate origin

**Figure 5:**
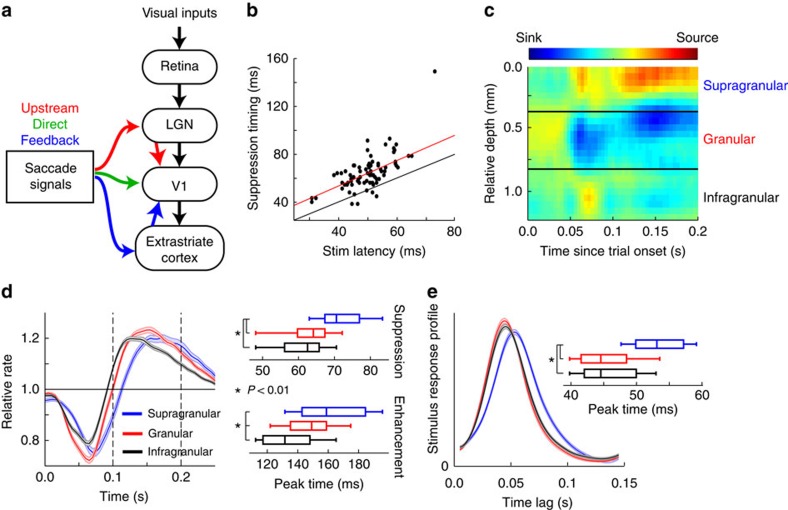
Timing and laminar profile of perisaccadic firing rate modulation suggests upstream origin. (**a**) Schematic showing three different sources of saccade-related modulatory signals: (red) signals targeting ‘upstream’ LGN inputs to V1; (green) direct modulatory inputs to V1; (blue) signals targeting downstream cortical structures that provide feedback projections to V1. (**b**) Perisaccadic firing rate suppression occurred significantly later for neurons with more delayed stimulus responses (Spearman’s rank correlation *ρ*=0.61; *P*=6.4 × 10^−9^; *n*=76). Red line shows linear fit and black line shows the diagonal. (**c**) Stimulus-onset-triggered current source density (CSD) profile from an example laminar probe recording. The location of a short-latency current sink is used to estimate the upper and lower boundaries of layer IV. (**d**) The time of maximal saccadic firing rate suppression was significantly delayed for multi-unit activity from supragranular electrodes (blue; *n*=58 across 9 recordings) compared with granular (red; *n*=55) and infragranular (black; *n*=85) electrodes. (**e**) Supragranular units also showed temporally delayed stimulus response profiles (see Methods).

Given that saccade-related extra-retinal signals have been observed in several different brain areas that project to V1, perisaccadic modulation in V1 could in principle arise from several different sources. We broadly classify these different sources as: (1) direct modulatory inputs, such as from the pulvinar; (2) signals inherited from ‘upstream’ (that is, from ‘feedforward’ geniculate inputs); and (3) feedback signals from extra-striate cortex (Fig. 5a). Importantly, these possibilities would result in different patterns of saccadic modulation timing across neurons. Specifically, if saccade modulation was inherited from the LGN, then it should be conveyed to V1 neurons by the same inputs that give rise to their stimulus processing. Thus, neurons with shorter-latency responses to the stimulus should also exhibit earlier saccadic suppression, whereas such a relationship would not be expected from a direct modulatory input to V1, or from signals generated by extra-striate feedback. Consistent with an upstream origin of the effects, we found that neurons that responded to the stimulus at shorter latencies (as determined by the timing of their stimulus filters; see Methods) also exhibited earlier post-saccadic firing rate suppression (Fig. 5b; *ρ*=0.61; *P*=6.4 × 10^−9^; *n*=76), as well as somewhat earlier firing rate enhancement (*ρ*=0.32; *P*=5.2 × 10^−3^).

A second key prediction of perisaccadic modulation arising from geniculate inputs is that its timing should have a laminar profile similar to that of the stimulus response latencies, with the earliest perisaccadic modulation occurring in the thalamo-recipient layers^38^,^39^. To test this, we estimated the locations of laminar boundaries in our linear multielectrode array recordings using current source density analysis, and classified the electrode contacts as ‘supragranular’, ‘granular’ or ‘infragranular’ (Fig. 5c; Methods). Indeed, we found significant laminar differences in the timing of perisaccadic firing rate suppression (*P*=2.0 × 10^−7^; two-way analysis of variance (ANOVA)) and enhancement (*P*=6.0 × 10^−9^; Fig. 5d) that matched the significant laminar differences in the timing of stimulus processing^40^,^41^ (Fig. 5e; *P*=2.2 × 10^−26^; two-way ANOVA). In particular, supragranular units exhibited both delayed saccade modulation and stimulus responses compared with granular and infragranular layers. Since the superficial layers of V1 are the predominant targets of inputs from both the pulvinar^42^ and from higher cortical areas^43^, this provides direct evidence against both of these potential sources of perisaccadic modulation.

### Perisaccadic changes driven by ‘upstream’ gain modulation

**Figure 6:**
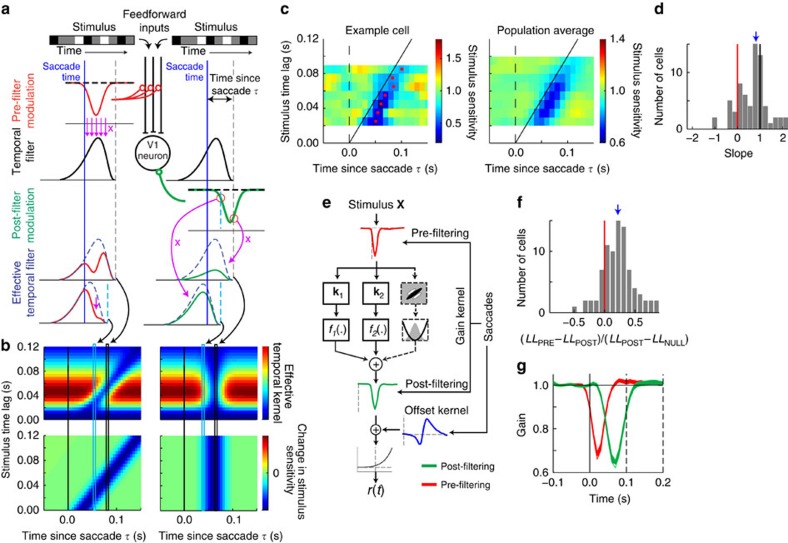
Perisaccadic changes in V1 stimulus processing are driven by upstream gain suppression. (**a**) Schematic illustrating the key differences between an ‘upstream’ source of gain modulation (left, red) and modulation arising from direct inputs to V1 (right, green). In the former (‘pre-filter’ modulation), perisaccadic suppression is already present in the stimulus-driven inputs to a V1 neuron, before application of its stimulus filters (illustrated here by a single temporal filter; black). Thus, the neuron’s firing rate at a delay *τ* after a saccade will be less sensitive to some stimuli, producing an altered ‘effective temporal kernel’ (shown for two example times; bottom left). Alternatively, direct perisaccadic suppression onto the V1 neuron, acting after temporal integration of its stimulus filters (‘post-filter’ modulation), will produce a uniform scaling of the neuron’s temporal kernel (bottom right). (**b**) The fundamental difference between these two sources of gain modulation is thus the pattern of perisaccaddic changes in the neuron’s sensitivity to stimuli at different latencies (bottom). (**c**) Perisaccadic changes in the neurons’ sensitivity to stimuli at different latencies (Methods); shown for an example neuron (left), and averaged across the population of neurons (right; *n*=84). The time of maximal perisaccadic suppression occurred systematically later for stimuli at larger latencies, consistent with the pre-filter suppression model. (**d**) The time of maximal saccadic suppression (red dots at left in **c**) systematically increased as a function of stimulus latency. Across the population, the slope of this relationship was much greater than 0 (median=0.82, blue arrow; *P*=3.3 × 10^−9^; *n*=67; see Methods), suggesting gain suppression occurs before the neurons’ temporal filtering. Red and black lines show slopes of 0 and 1, respectively. (**e**) Schematic of the stimulus processing model with perisaccadic gain suppression incorporated either upstream of the stimulus filters (red) or after integration of subunit inputs (green). (**f**) The pre-filter gain model performed significantly better than the post-filter gain model (median relative *LL* improvement=22%, blue arrow; *P*=2.3 × 10^−10^; *n*=84; Methods). (**g**) Average pre-filter gain kernels (red), showing that the gain acting on stimuli presented over a short window following saccade onset was sharply suppressed. By comparison, post-filter gain kernels (green) were temporally delayed relative to the pre-filter model, reflecting the delay associated with processing by the stimulus filters.

These different possible sources of gain modulation are also expected to produce qualitatively different changes in the stimulus processing of individual neurons. In particular, while our analysis thus far has treated perisaccadic gain modulation as acting on the integrated total stimulus-driven input at each time (such as would be generated by a direct modulatory signal), an ‘upstream’ origin of saccadic suppression would result in modulation of a neuron’s stimulus-driven inputs before they are temporally integrated (Fig. 6a). Thus, such upstream, or ‘pre-filter’, gain suppression would cause the neuron to be less sensitive to stimuli presented at particular times following a saccade (effectively reshaping the neuron’s ‘temporal filter’). In contrast, ‘post-filtering’ gain modulation will suppress the neuron’s response to the temporally integrated set of stimulus-driven inputs at a given time, effectively scaling the entire temporal kernel uniformly without changing the relative contribution of stimuli at different latencies. Thus, the fundamental test to differentiate between upstream and direct gain modulation is whether a neuron’s sensitivity to stimuli at different latencies exhibits systematic perisaccadic changes (Fig. 6b).

We directly tested for such changes by measuring the timing of perisaccadic modulation of the neurons’ sensitivity to stimuli at different latencies (Methods). Neurons’ selectively showed reduced sensitivity to stimuli that occurred shortly after the onset of a saccade, rather than becoming uniformly less responsive to the entire stimulus history (Fig. 6c), which is consistent with the source of gain suppression being upstream of V1. To quantify this effect for each neuron, we computed the slope of the relationship between the stimulus latency, and the timing of maximal saccadic suppression. Across the population of neurons these slope values were consistently near 1 (median=0.82; *n*=67; Fig. 6d; Methods), as predicted by the pre-filtering gain suppression model (with any noise tending to reduce the slope estimates). In contrast, these results are clearly inconsistent with the post-filtering gain suppression model (*P*=3.3 × 10^−9^), which would predict a slope of zero.

This suggests that a model of V1 processing that explicitly incorporates ‘pre-filter’ gain changes (that is, acting before the subunit filters) should provide a better description of perisaccadic responses than our previous models (for example, Fig. 2), which have treated gain modulation as acting on a neuron’s integrated inputs (‘post-filter’). We thus tested this by directly comparing models where perisaccadic gain modulation acted either before or after application of a neuron’s stimulus filters (Fig. 6e). Consistent with the measurements above, the ‘pre-filtering’ gain suppression models performed significantly better than the ‘post-filtering’ models (Fig. 6f; *P*=2.3 × 10^−10^). Furthermore, the timing of such ‘upstream’ gain suppression was well synchronized with saccade onset (Fig. 6g), and its duration was consistent with that of the saccades themselves (average saccade duration=36 ms), demonstrating that saccades selectively suppress the effects of stimuli presented during the saccades. This ‘upstream’ model also provided a significantly better description of microsaccadic gain suppression (*P*=1.9 × 10^−5^), further highlighting the qualitative similarity of modulation produced by saccades and microsaccades.

### Saccade modulation is largely due to extra-retinal signals

**Figure 7:**
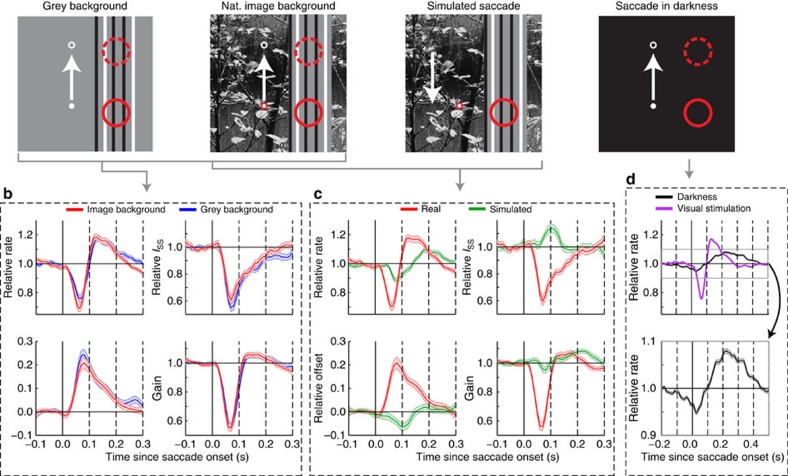
Saccade modulation is due largely to extra-retinal signals. (**a**) Schematic diagrams showing three different saccade conditions. Left: saccades made with a grey background. Middle left: saccades made with natural image backgrounds. Middle right: simulated saccades generated by translating the background image during static fixation. Right: saccades made in total darkness. (**b**) Comparison of modulation by saccades made with image backgrounds (red) versus grey backgrounds (blue). Clockwise from top-left, population averages (*n*=64) of perisaccadic firing rate, *I*_*SS*_, gain and offset. Saccades with image backgrounds produced similar modulation of average rates, with slightly stronger suppression (median=1.22-fold; *P*=8.9 × 10^−7^), but equivalent enhancement (*P*=0.30). They also produced similar, but slightly weaker, reductions of *I*_SS_ (median=0.86-fold; *P*=2.6 × 10^−3^). (**c**) Similar to **b**, comparing the effects of real (red) and simulated (green) saccades (*n*=56). Simulated saccades produced biphasic firing rate modulation, though both suppression and enhancement were weaker (suppression: 0.52-fold, *P*=8.4 × 10^−11^; enhancement: 0.57-fold, *P*=8.9 × 10^−9^), and temporally delayed (suppression: 1.31-fold, *P*=1.2 × 10^−5^; enhancement: 1.48-fold, *P*=3.0 × 10^−8^). Simulated saccades produced qualitatively different effects on gain and offset, such that they produced a slight increase in *I*_SS_ resulting from a reduction, rather than increase, in firing rate offset. (**d**) Top: average relative MU firing rates showed biphasic modulation following saccades made in complete darkness (*n*=96 MUs). Compared with saccade modulation during visual stimulation (magenta), saccades made in darkness (black) produced weaker modulation (particularly suppression), and a more prolonged period of post-saccadic enhancement (note wider time axis). Bottom: expanded view of the region highlighted by horizontal lines above.

There are two potential sources for saccade modulation originating upstream of V1: extra-retinal signals targeting the LGN^11^,^12^,^13^, or saccade-generated effects within the retina itself. While our stimulus and behavioural task were designed to minimize any saccade-induced changes to the visual stimulus in and around the neurons’ RFs (Fig. 1a), it remains possible that retinal signals from outside the display (that is, the far surround) contribute to the observed saccade modulation, similar to the so-called ‘periphery’, or ‘shift-effect’ observed in the retina^44^,^45^,^46^,^47^. To examine this possibility, we tested whether modulation of V1 activity was different when saccades were made with static natural images in the background, in place of uniform grey (Fig. 7a). Saccades made with an image background produce much larger changes in the retinal stimulus outside the neurons’ RFs compared with those made with a grey background. Thus, if wide-field retinal signals were responsible for the observed effects, we would expect to see substantially stronger modulation by saccades made on an image background. However, the presence of an image background had a small impact on perisaccadic firing rate modulation, producing slightly stronger firing rate suppression (median: 1.22-fold) compared with a grey background, and no difference in firing rate enhancement (Fig. 7b). Furthermore, the saccadic suppression of stimulus selectivity was very similar in the two conditions, being slightly weaker (0.86-fold) in the presence of an image background (Fig. 7b).

To further test the effects of wide-field retinal signals, we simulated the retina-mediated effects of saccades by rapidly translating the same background images while the animals maintained fixation on a static target (Fig. 7a). While these ‘simulated saccades’ produced a biphasic firing rate modulation, the magnitude of both suppression and enhancement were substantially smaller, and temporally delayed, relative to that evoked by real saccades (Fig. 7c). Furthermore, simulated saccades produced qualitatively different effects on stimulus selectivity, leading to a slight increase in *I*_*SS*_, rather than the pronounced decrease produced by real saccades (Fig. 7c). Thus, while the existence of firing rate modulation by simulated saccades shows that saccade-driven retinal signals in the far surround can affect V1 responses, such effects cannot account for the quantitative or qualitative changes we observe following saccades. Our results therefore suggest that perisaccadic modulation is primarily driven by extra-retinal signals.

The presence of extra-retinal signals associated with saccades is also suggested by the modulation of V1 neuron firing rates by saccades made in darkness^2^,^3^,^4^,^5^. We thus measured spontaneous multi-unit (MU) activity while one animal freely made saccades in darkness, taking care to ensure that all sources of light had been eliminated from the recording room. Consistent with previous studies^2^,^3^,^4^,^5^, we found clear, though weak, biphasic modulation of V1 activity by saccades in darkness (*n*=96 MUs; Fig. 7d). While these results confirm the presence of extra-retinal signals in V1, they are not directly comparable to the saccade modulation we demonstrate here, as there was no ‘stimulus processing’ for saccades to modulate. Further, the baseline firing rates in darkness were markedly lower such that the relatively weaker suppression of firing rates might be due to ‘floor effects’. The much weaker effects of saccades on spontaneous activity highlight the importance of studying saccadic modulation in the context of stimulus processing, which not only allows for a much more sensitive probe of such modulation but can also reveal qualitatively distinct effects.

## Discussion

Our results show that saccades produce clear modulation of visual processing in V1. This is consistent with previous work showing extra-retinal modulation of V1 spontaneous activity^2^,^3^,^4^,^5^, but appears in contrast with a range of previous studies that found minimal effects of saccades on visually evoked responses^5^,^18^,^19^,^20^,^21^,^22^. (Note that while Ruiz *et al.*^22^ did show clear differences in the responses of V1 neurons to saccade-induced and flashed stimuli, most of the effect they observed was due to saccade-driven visual stimulation in the neurons’ surround, rather than extra-retinal effects like those shown here.) This discrepancy is likely due to the fact that previous studies attempted to measure perisaccadic modulation by comparing a neuron’s response to a stimulus that was either introduced into its RF by a saccade or flashed into (or swept over) its RF. Such direct comparisons will generally be confounded by uncontrolled variability in the retinal stimulus introduced by saccades. The stimuli used in these previous studies also evoke large changes in firing rate that are intermixed with any modulatory effects of saccades, adding to the difficulty of disentangling saccade-driven from stimulus-driven effects. In contrast, using a dynamic noise stimulus whose properties are independent of saccades^26^, we are able to precisely gauge the dynamics of saccade modulation in an unbiased manner.

The implicit coupling of eye movements with changes in the retinal stimulus has also greatly confounded studies of microsaccadic modulation, which can produce a variety of disparate effects^5^,^26^,^28^,^29^,^30^. Our finding that microsaccades produce a robust biphasic modulation of average firing rates is consistent with the results of Hass *et al.*^26^, who also used a dynamic noise stimulus, and is broadly consistent with recent work showing microsaccadic suppression^48^. We further show that microsaccades and saccades produce a similar set of changes to V1 stimulus processing, although microsaccades produce substantially weaker reductions in stimulus selectivity. This qualitative similarity fits with recent work suggesting that, rather than being distinct behaviours, microsaccades form part of a continuum of saccadic eye movements^35^,^36^.

A key innovation of our study was the use of sophisticated models of V1 neuron stimulus processing. Such models allowed for analysis of the detailed interactions between saccade-driven and stimulus-driven inputs, rather than simply measuring the effects of saccades on average firing rates. We were thus able to determine that biphasic firing rate modulation by saccades was generated by an overlapping combination of gain suppression and a stimulus-independent increase in firing rate ‘offset’. The combined effect of these changes was a monophasic reduction in stimulus selectivity lasting throughout the period of perisaccadic firing rate modulation. This reduction in stimulus selectivity was not predictable from the observed changes in firing rate alone, emphasizing the potential limitations of inferring changes in stimulus processing from average firing rates.

We also utilized a number of detailed analyses, coupled with experimental controls, to gain insight into the source of perisaccadic modulation in V1. First, while our experimental design minimized any saccade-driven changes to the visual stimulus in and around the neurons’ RFs (Supplementary Fig. 2), we also examined the contribution of saccade-driven visual stimuli in the far surround. We found that while such far surround stimuli did modulate V1 firing rates, the effects were substantially weaker, and temporally delayed, compared with the extra-retinal effects of saccades. Further, visual stimuli in the far surround did not produce any reduction of stimulus selectivity, and hence could not explain the marked changes in stimulus processing elicited by saccades. In fact, while stimuli far outside a neuron’s RF have been shown to modulate the activity of retinal ganglion cells in many species^44^,^45^,^46^,^47^, such effects are substantially weaker in the primate^47^,^49^. We cannot eliminate the possibility that transient structural changes in the eyes caused by saccades, such as bending of cone receptors^50^ or wobbling of the lens^51^ produce suppression of visual responses, although such effects could not explain the modulation of spontaneous activity by saccades in total darkness^2^,^3^,^4^,^5^.

While extra-retinal signals associated with saccades have been observed in several different areas that provide inputs to V1, including the LGN^11^,^12^,^13^, pulvinar^52^,^53^, and downstream cortical areas^6^,^8^,^9^, we presented several lines of evidence strongly suggesting that the observed perisaccadic modulation in V1 is inherited from the LGN. First, the timing of saccadic suppression across cortical lamina matched the timing of stimulus responses, with both occurring earliest in the thalamo-recipient layers and latest in the superficial layers (Fig. 5d,e). This is in stark contrast to the pattern expected from pulvinar or extra-striate inputs, which predominantly terminate in the superficial layers^42^,^43^. Second, the timing of perisaccadic firing rate modulation was strongly correlated with the latency of a neuron’s stimulus response (Fig. 5b), suggesting the saccadic suppression signal is conveyed by each neuron’s stimulus-driven inputs. In order for pulvinar or extra-striate inputs to produce this pattern they would have to target V1 neurons with variable latencies tied to each neuron’s particular stimulus response timing: an extremely unlikely projection pattern. Finally, we showed that the functional form of perisaccadic modulation in V1, with systematic perisaccadic changes in each neuron’s temporal processing of the stimulus (Fig. 6), is only consistent with an ‘upstream’ origin of the effect.

Our work also highlights the nontrivial changes that can result from relatively simple modulation of feedforward signals interacting with nonlinear spatiotemporal stimulus processing. Indeed, the propagation of saccadic suppression through multiple nonlinear processing stages could produce the distinct modulatory effects that have been observed at different levels of the visual hierarchy^6^. Furthermore, the synchronous suppression of visual inputs to V1 during saccades could trigger a variety of changes in the local network dynamics, such as resetting of ongoing oscillations^4^, or modulation of contrast-gain control mechanisms^54^,^55^. In fact, the observed stimulus-independent increase in firing rates elicited by saccades might arise from a suppression of contrast normalization mechanisms within V1 triggered by the synchronous reduction of stimulus-driven inputs.

Although our analyses identified several specific perisaccadic changes in V1 stimulus processing, more general forms of saccade modulation, such as changes in the neurons’ spatial tuning, remain possible. While we cannot completely rule out more general changes, several lines of evidence suggest that our models are not missing important elements of perisaccadic stimulus processing. First, we computed spike-triggered average (STA) stimuli at each latency relative to saccade onset. Neurons with clear structure apparent in their ‘saccade conditional STAs’ showed only a post-saccadic decrease in the STA amplitude, with no discernable changes in spatial tuning (Supplementary Fig. 5). We also estimated ‘subspace’ models where the filters characterizing a neuron’s stimulus selectivity were allowed to vary as a function of time relative to saccade (Methods). Despite the much broader range of possible perisaccadic modulation represented by these models, they also showed a very similar monophasic suppression of stimulus selectivity following saccades (Supplementary Fig. 6). Thus, our analysis has likely identified at least the dominant perisaccadic changes in stimulus processing.

It is tempting to interpret the perisaccadic gain suppression observed here as a neural correlate of the reduced perceptual sensitivity to perisaccadic visual stimuli^10^,^56^. However, several observations argue against this interpretation. First, perceptual suppression is thought to be weak for the high spatial frequencies considered here^24^,^25^,^57^. Second, while perceptual suppression is isolated to the magnocellular pathway^25^, saccadic modulation has been observed in both parvocellular and magnocellular LGN neurons^11^,^13^, and microsaccadic modulation is similar in both cone-opponent and non-opponent V1 neurons^26^. Finally, perceptual suppression has been shown to start significantly before saccade onset, while we did not observe suppression of V1 responses to stimuli occurring prior to saccade onset (for example, Fig. 6g). Taken together, these results thus support the notion that there are multiple pathways of extra-retinal signalling, rather than a single modulatory signal that targets the LGN and propagates throughout the visual hierarchy^10^. One possibility is that direct projections from the inferior pulvinar to area MT^53^ are responsible for mediating perisaccadic perceptual suppression, while separate projections from the superior colliculus to the LGN^58^ give rise to the perisaccadic modulation observed here in V1.

A functional role for the stimulus-independent post-saccadic firing rate increase is difficult to ascertain. While previous studies have suggested that post-saccadic increases in visual neuron firing rates might reflect a window of enhanced processing of the stimuli introduced by saccades^9^,^13^, our finding that these firing rate increases are accompanied by decreased stimulus selectivity argues against this. If perisaccadic modulation in V1 serves to reset processing in the cortical network (such as through ‘phase-resetting’ of ongoing network oscillations^4^), such synchronized firing rate modulations may help to parse stimuli presented during separate fixations^59^, and ultimately integrate them into a seamless visual percept.

## Methods

### Electrophysiology

Multielectrode recordings were made from primary visual cortex (V1) of two awake head-restrained male rhesus macaques (Macaca mulatta; 13–14-year old). We implanted a head-restraining post and scleral search coils under general anaesthesia and sterile conditions^60^. Animals were trained to fixate for fluid reward. In one animal, we implanted a 96-electrode planar ‘Utah’ array (Blackrock Microsystems; 400 μm spacing). In the second animal, we implanted a recording chamber and used a custom microdrive to introduce linear electrode arrays (U-probe or V-probe, Plexon; 24 contacts, 50 μm spacing) on each recording day. Eye position was monitored continuously using scleral search coils, sampled at 600 Hz. Stimuli were displayed on cathode ray tube monitors (100 Hz refresh rate) subtending 24.3 × 19.3° of visual angle, viewed through a mirror haploscope. All protocols were approved by the Institutional Animal Care and Use Committee and complied with Public Health Service policy on the humane care and use of laboratory animals.

Broadband extracellular signals were sampled continuously at 30 or 40 kHz and stored to disk. Spikes were detected using a voltage threshold applied to the high-pass filtered signals (low cutoff frequency 100 Hz). Thresholds were set to yield an average event rate of 50 Hz, and were subsequently lowered if needed to capture any putative single unit (SU) spiking. Spike sorting was performed offline using custom software. Briefly, spike clusters were modelled using Gaussian mixture distributions that were based on several different spike features, including principal components, voltage samples and template scores. The features providing the best cluster separation were used to cluster SUs. Cluster quality was quantified using a variety of measures including ‘L-ratio’, ‘isolation distance’^61^, as well as a variant of ‘d-prime’. Only spike clusters that were well isolated using these measures—confirmed by visual inspection—were used for analysis. We verified that our results were not sensitive to cluster isolation.

### Behavioural task and visual stimuli

During experiments the animals performed a simple fixation task, whereby they were required to maintain gaze within a small window around a fixation target to obtain a liquid reward after each completed 4-s trial. The fixation target made periodic jumps (3–4° amplitude) parallel to the orientation of the bar stimuli (Fig. 1a) requiring the animal to make ‘guided saccades’ within 300 ms to maintain fixation on the target. Guided saccades alternated between going outward from the central fixation point, and returning to the central fixation point, repeating this pattern in alternating directions.

During this time a ternary bar noise stimulus was presented, consisting of random patterns of black, white and grey bars (matching the mean luminance of the screen). Bars stretched the length of the monitor, and spanned a region from 1 to 4° wide, centred on the neurons’ RFs. Individual bars had a width of 0.057° for the Utah array recordings, and ranged from 0.038 to 0.1° for the linear array recordings, depending on the RF sizes of the recorded neurons. Bar patterns were displayed at 100 Hz, and were uncorrelated in space and time. The probability of a given bar being non-grey (that is, black or white) was set to a sparse value in most experiments (88% grey), although in several recordings we used a dense distribution (33% grey), yielding similar results. For the linear array recordings, the orientation of the bars was chosen to correspond to the preferred orientation of the majority of units recorded in a given session. In cases where the neurons did not have a consistent preferred orientation (including the planar array recordings), we performed the experiments with two different bar orientations (vertical and horizontal).

In some trials, we displayed histogram-equalized natural images^62^ in the background. In a subset of these trials, we simulated the effects of saccades on the background images by rapidly translating the images (according to the average trajectory of measured saccades) while the animal fixated a static target. In one animal, we also measured V1 activity during free viewing in complete darkness, taking care to ensure that all sources of light had been eliminated from the recording room.

### Criteria for data selection

The first 200 ms and last 50 ms of each trial were excluded from analysis to minimize the impact of onset transients and fixation breaks, and trials lasting <750 ms were excluded entirely.

We only used SUs that had average firing rates during stimulus presentation of at least 5 spikes per second. We also excluded one SU for which the stimulus processing model did not perform better than the null model (predicting only the average rate; see below). For analysis of saccade (microsaccade) modulation, we only used units for which there were at least 500 saccades (microsaccades). When comparing different saccade conditions (Fig. 7), we required at least 250 saccades of each type (for example, with image and grey backgrounds). For neurons that were recorded with multiple stimulus orientations, we used the stimulus orientation for which the neurons’ stimulus processing models had a larger log-likelihood improvement relative to the null model. We verified that all results were robust towards the precise choices of these selection criteria.

### Saccade detection

Saccades were detected by finding times when the instantaneous eye velocity (measured by the eye coils) exceeded 10° s^−1^, with the onset of saccades defined as the time when eye velocity first exceeded 3° s^−1^. We found that brief high-frequency artefacts in the coil signals following saccades could bias identification of the precise saccade end points. Thus, we detected saccade end points using slightly higher eye velocity thresholds of 10° s^−1^ (for saccades) and 5° s^−1^ (for microsaccades) to obtain more reliable estimates of saccade/microsaccade durations. Blinks were identified by finding instances when any component of the instantaneous eye velocity exceeded a threshold (three times the median speed) for at least 100 ms.

‘Guided saccades’ were defined as saccades whose amplitude along the direction of the fixation target jump was greater than half the magnitude of the target jump. Microsaccades were defined as saccades whose amplitude was <1° (ref. 28). One of the animals tended to make rapid bursts of several microsaccades at frequencies around 10 Hz. Although we confirmed that microsaccades within a burst produced similar modulation of V1 activity, we nevertheless excluded them to minimize the impact of such rhythmic microsaccade timing on our triggered average analysis. We thus excluded microsaccades that occurred within 150 ms of another saccade. We classified microsaccades as either ‘parallel’ or ‘orthogonal’ to the stimulus orientation (Supplementary Fig. 2d) based on which direction was closer to the direction of the microsaccade (measured using the eye coils). Similarly, saccade accuracy (Supplementary Fig. 2c) was determined by measuring the eye position displacement orthogonal to the bar stimuli.

### Model-based eye tracking

Since V1 neurons in and around the fovea are sensitive to fine spatial detail of the stimulus, we used a recently developed method for precisely tracking the animals’ fixational eye movements^31^. This method utilizes probabilistic models of each neuron’s stimulus processing to infer the most likely sequence of eye positions (orthogonal to the bar stimuli) given the spiking activity recorded from a population of V1 neurons (using multi- and single-unit activity). The inferred sequence of eye positions is then used to reconstruct the retinal stimulus. As described previously, we used a leave-one-out cross-validation procedure such that whenever analysing the stimulus processing of a given neuron, we used eye positions inferred from the population excluding that unit.

While using this method to precisely track eye position substantially improved the stimulus processing models—and thus improved our ability to resolve detailed elements of saccade modulation—our results for saccade modulation remained qualitatively similar without using this technique (assuming perfect fixation along the direction orthogonal to the bar stimuli). Correcting for the animals’ fixation error was important for obtaining unbiased analyses of the effects of microsaccades, however, as they tended to be ‘corrective’ (that is, they systematically reduced fixation error; Supplementary Fig. 4)^63^,^64^.

### Analysis of the effects of saccades on stimulus statistics

To calculate the power spectrum of the stimulus on the retina during saccades, we incorporated the measured intrasaccadic displacement of the stimulus using the average eye velocity profile in the dimension orthogonal to the bar stimuli: *v*_orth_(*τ*). We estimated this according to: *v*_orth_(*τ*)=*v*_tot_(*τ*)sin^−1^*θ*, where *τ* is the time relative to saccade onset, *θ* is the measured angle of the saccade relative to the bar stimuli and *v*_tot_(*τ*) is the total eye speed profile. Compared with direct measurements of *v*_orth_(*τ*), this estimate is less sensitive to small artefacts in the coil signals during saccades, and is equivalent to assuming straight saccade trajectories. However, we obtained similar results when using the direct measurements of *v*_orth_(*τ*).

To quantify the effects of saccades, we averaged together the stimulus power spectra during saccades where the orthogonal displacement was in either direction (for example, leftward and rightward motion for vertical bar stimuli). We then compute the relative change of the intrasaccadic amplitude spectrum: 
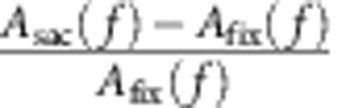
. As a function of temporal frequency *f*, evaluated at the preferred spatial frequency of each recorded SU (derived from peaks of the Fourier transform of the neurons’ spatiotemporal stimulus filters). For this calculation, we used a bar width of 0.057° and a sparsity of 88% grey, which were the stimulus parameters used in the majority of our experiments. The cathode ray tube phosphor luminance response was measured using a photodiode and oscilloscope.

### Estimation of stimulus processing models

We used a recently developed modelling framework to describe each neuron’s stimulus processing as a second-order linear-nonlinear cascade (LNLN)^32^,^33^,^65^,^66^. Specifically, a neuron’s predicted spike rate *r*(*t*) is given by a sum over LN subunits, followed by a spiking nonlinearity function:




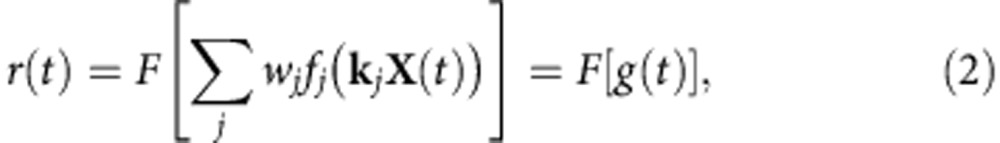




where *g*(*t*) is the ‘generating signal’, **X**(*t*) is the retinal stimulus at time *t* (with relevant history ‘time-delay-embedded’), the **k**_***j***_ are a set of stimulus filters, with corresponding static nonlinearities *f*_*j*_(.), the *w*_*j*_ are coefficients that determine whether each input is excitatory or inhibitory (constrained to be ±1) and *F*[.] is the spiking nonlinearity function. Here we use a spiking nonlinearity of the form:




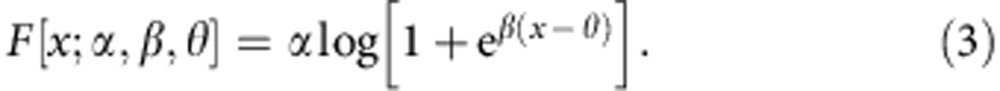




Assuming the neuron’s spike counts *R*_obs_(*t*) are described by a conditionally inhomogeneous Poisson count process, the log-likelihood *LL* of the model is given by:




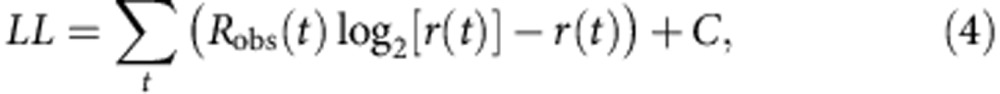




where *C* is independent of the predicted rate *r*(*t*), and thus unaffected by changes in model parameters. Estimation of model parameters is accomplished by direct maximization of the *LL*^33^.

While the set of functions *f*_*j*_(.) can be inferred from the data^33^,^65^,^66^, in this case we limit the *f*_*j*_(.) to be linear and squared functions, which represents a (low-rank) quadratic approximation of the neuron’s stimulus response function that provides a robust description of the stimulus processing of V1 simple and complex cells^32^,^33^.

To determine the number of excitatory and inhibitory quadratic subunits, we fit a sequence of such models with increasing numbers of subunits. We stopped adding subunits when the cross-validated model *LL* (using a randomly selected subset of 20% of trials for cross-validation) no longer improved, allowing up to a maximum of 3 excitatory and 3 inhibitory ‘squared’ subunits in addition to the linear filter.

As a final step, we then ‘split’ the linear filter of the quadratic model into two separate threshold-linear subunits, one excitatory and one inhibitory, and re-estimated the model parameters. This splitting of the linear subunit allowed for unambiguous segregation of inputs into either excitatory or inhibitory components, but otherwise provided very similar results to the pure quadratic models.

To reduce overfitting, we incorporated spatiotemporal smoothness and sparseness regularization when estimating the stimulus filters. Details regarding stimulus filter regularization, as well as methods for finding maximum likelihood parameter estimates, are described in McFarland *et al*^33^.

### The nonparametric saccade modulation model

To estimate the firing rate as a ‘nonparametric’ function of the generating signal *g* and the time since saccade onset *τ*, we represented the two-dimensional response function *r*(*g*,*τ*) as a linear combination of piecewise-linear basis functions. Specifically, we first created a rectangular grid segregating the domain into bins (equi-populated in *g*, and uniform in *τ*), and used this grid to create a uniform triangulation of the domain. This triangulation then defined a set of local piecewise-linear basis functions, one associated with each vertex in the grid, such that the value of the basis function *ψ*_*k*_ associated with the *k*th vertex is 1 at the *k*th vertex and 0 at all other vertices. These basis functions thus resemble a set of ‘pyramids’ centred on each vertex.

Our model for the response function *r*(*g*,*τ*):




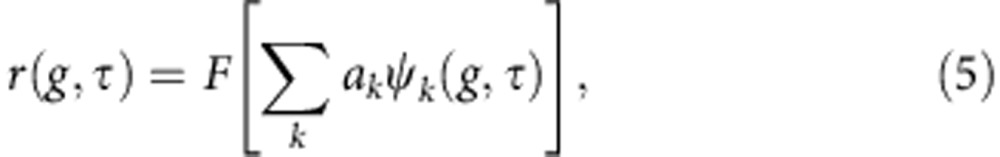




is then linear in the coefficients *a*_*k*_ weighting each of the basis functions, and can be estimated using standard procedures. When estimating the *a*_*k*_ we also incorporate smoothness regularization (that is, an L2 penalty on the Laplacian of the *a*_*k*_ with respect to the grid coordinates). Note that the term ‘nonparametric’ here refers to the fact that these models do not assume a particular parametric form for the dependence of *r* on *g* and *τ*.

### The perisaccadic ‘gain/offset’ model

We modelled the effects of multiplicative and additive saccade modulation by incorporating linear ‘gain’ and ‘offset’ kernels *α*(*τ*) and *c*(*τ*), respectively, whose contribution is given by convolution with the vector of binned saccade times *s*(*t*):




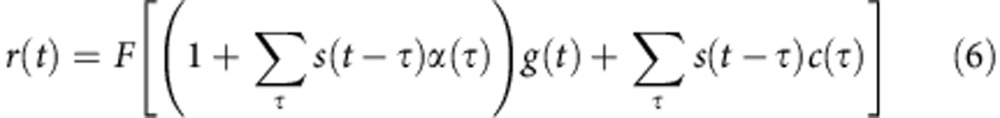




To estimate these ‘internal’ gain and offset functions, we utilized a set of saccade indicator functions *I*_*τ*_(*t*), taking a value of one if a saccade occurred with latency *τ* at time *t*. The model then becomes:




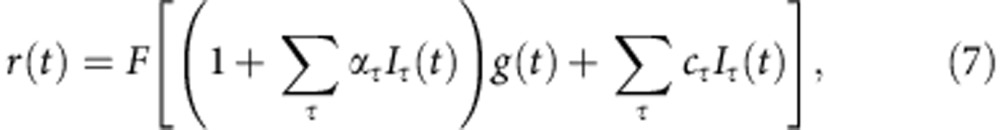




which for a given *g*(*t*) is linear in the coefficients *α*_*τ*_ and *c*_*τ*_, making parameter estimation straightforward

Note that the gain/offset model, unlike the nonparametric model, naturally handles the case when multiple saccades occur nearby in time (as is often the case with microsaccades), by assuming linear convolution of saccade times with the gain and offset kernels. Furthermore, because the gain/offset model produces similar results to the nonparametric model using many fewer parameters (Supplementary Fig. 3), we use this simpler model for subsequent analysis of perisaccadic modulation.

### The upstream gain model

When modelling perisaccadic gain modulation occurring ‘upstream’ of the V1 neuron’s stimulus processing, we define a gain kernel *γ*(*τ*) acting on the stimulus **X**(*t*) directly (that is, *γ*(*τ*) determines the gain associated with stimuli that were presented at a latency *τ* relative to saccade onset). To estimate *γ*(*τ*), we introduce a set of saccade indicator matrices **J**_*τ*_(*t*) giving:




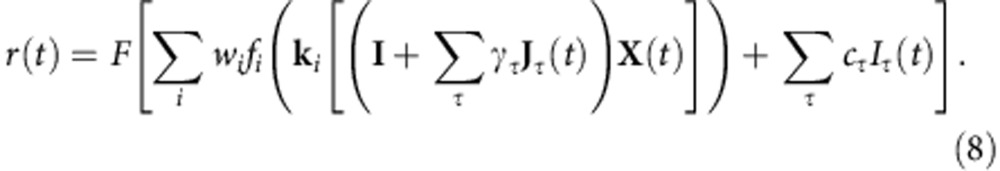




Specifically, if the time-embedded stimulus representation **X**(*t*) has dimensionality *d*, then the **J**_τ_(*t*) are a set of *d* × *d* matrices specifying whether each element of **X**(*t*) occurred at latency *τ* relative to a saccade.

For a given set of stimulus filters **k**_i_, the problem of maximizing the model *LL* with respect to the coefficients *γ*_*τ*_ is then well behaved, essentially becoming equivalent to the problem of estimating the stimulus filters themselves^33^.

### Comparing upstream and direct gain suppression models

To measure perisaccadic changes in a neuron’s sensitivity to particular stimulus latencies, we first created a set of stimulus processing models that used only the values of the stimulus at a single latency to predict a neuron’s response. Specifically, for each stimulus latency *L*, we modelled the neuron’s response *r*(*t*) as a function of the stimulus **s**(*t*−*L*) using the same ‘LNLN cascade’ model structure as above (equation (2)), but where the stimulus filters of each subunit acted only on the stimulus at latency *L*. To determine these stimulus filters, we simply used the corresponding spatial slices of the full spatiotemporal stimulus filters for each neuron, and only re-estimated the weights associated with each subunit input. However, we found similar results when estimating these spatial filters at each latency directly.

For each model (corresponding to a particular latency), we then estimated perisaccadic ‘gain’ and ‘offset’ kernels (as in equation (6)). The set of estimated gain kernels *α*_*L*_(*τ*) were then used to measure the neuron’s sensitivity to stimuli at each latency *L*, and time relative to saccade onset *τ* (Fig. 6c). Note that because the ensemble of stimuli is virtually identical at each time lag relative to saccade onset (Supplementary Fig. 2), there is no *a priori* reason to expect different timing of perisaccadic suppression for stimuli at different latencies. Rather, the existence of such a relationship is a clear indication that gain suppression occurs before the spatiotemporal stimulus processing generating the neuron’s response.

Because estimates of perisaccadic changes in stimulus sensitivity were unreliable for stimulus latencies to which a neuron was not responsive, we excluded (from all analysis) stimulus latencies that did not sufficiently modulate a neuron’s response. We determined the range of latencies that a neuron was responsive to by measuring the coefficient of variation (ratio of the s.d. to the mean) of model-predicted firing rates at each latency, and excluded latencies where the coefficient of variation was below a fixed threshold. Our results were insensitive to the precise choice of this threshold, and the relationship between suppression timing and stimulus latency remained significant (*P*=2.8 × 10^−7^; *n*=84) even in the absence of any such threshold (using all stimulus latencies from 0 to 150 ms).

To calculate the relationship between saccadic suppression timing and stimulus latency, we first found the time of maximal suppression *τ*_max_ at each latency (given by the minimum of *α*_*L*_(*τ*) in the range from 0 to 200 ms after saccade onset). The slope of *τ*_max_ as a function of latency *L* was then estimated using robust regression. We only included slope estimates for neurons where at least five stimulus latencies were usable (*n*=67 out of 84 SUs). Furthermore, in the population average analysis of Fig. 6c, we only included stimulus latencies where there were at least 20 SUs contributing to the average.

### The subspace models

Although completely general perisaccadic changes to the form of *g*(**X**) require estimation of an intractably large number of parameters, it is reasonable to expect that any changes in stimulus tuning will occur within the stimulus subspace spanned by the set of stimulus filters **k**_***i***_ of each model’s ‘subunit inputs’. If Γ=**KX** represents the projection of the stimulus onto the vectors **k**_***i***_ (constituting the columns of the matrix **K**), we can then measure how saccades modulate the relative contributions of these different stimulus dimensions by fitting models of the form:




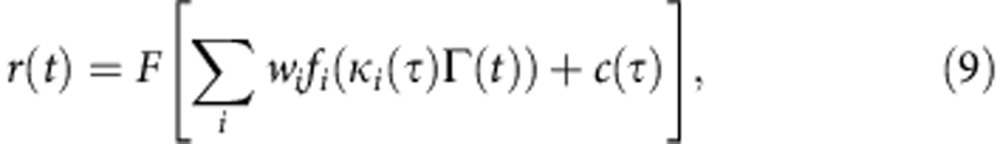




where the set of stimulus filters *κ*_*i*_(*τ*) at each time relative to saccade onset now represent linear combinations of the ‘basis filters’ **k**_i_. Each stimulus filter *κ*_*i*_(*τ*) is then characterized by *n* × *L* parameters, where *n* is the number of basis vectors and *L* is the number of perisaccadic time lags considered. Estimation of the *κ*_*i*_(*τ*) can be accomplished using a set of indicator functions *I*_*τ*_(*t*) as described above:




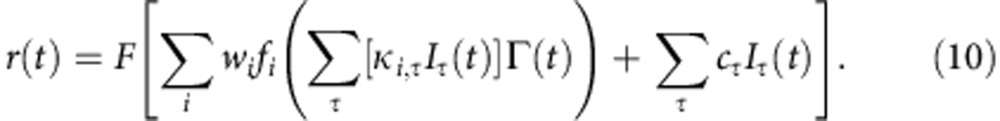




When estimating the subspace models, we utilized a simple quadratic model structure consisting of a linear filter and two (excitatory) squared filters (Supplementary Fig. 6).

### Regularization of perisaccadic kernels

When estimating perisaccadic gain and offset kernels, we incorporated temporal smoothness regularization (that is, a penalty on the second derivative of the coefficient vectors with respect to the time lag *τ*). We selected the regularization strengths (‘hyperparameters’) heuristically to provide slightly smoothed kernels, using the same values for all neurons (as well as when comparing pre- and post-filtering gain suppression models). We also scanned a large range of regularization strengths and verified that our results were robust to this choice.

### Response gain, offset and single-spike information

To quantify perisaccadic changes in a neuron’s ‘baseline’ response to the generating signal *r*_0_(*g*), we estimated the multiplicative ‘gain’, and additive ‘offset’, at each time *τ* as: *r*(*g*,*τ*)=*a*(*τ*)*r*_0_(*g*)+*c*(*τ*), using linear regression. We normalize the perisaccadic response gain and offset functions by their average values over time lags before saccade onset.

The single-spike information (*I*_SS_) provides a measure of the information conveyed in a neuron’s response *r*(**X**) about the stimulus **X** (in bits per spike), and is given by:




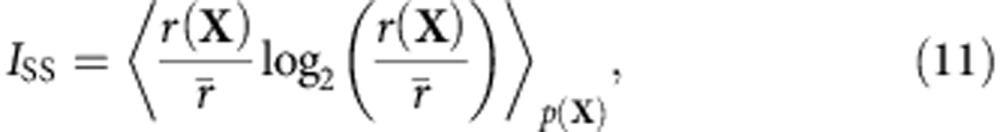




which can be approximated with a time average:




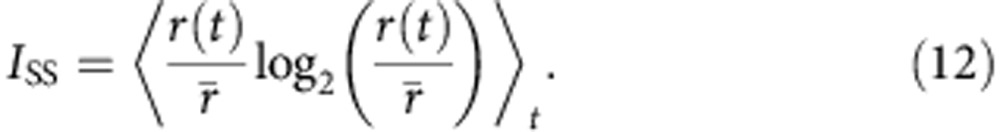




*I*_SS_ is a commonly used approximation for the mutual information between the stimulus **X**(*t*) and binned spike response *R*_obs_(*t*), which is valid in the limit of small time bins^34^. To estimate *I*_SS_ for a model-predicted firing rate *r*(*t*) as a function of time relative to saccades, we simply average over the set of times where a saccade occurred at latency *τ*:




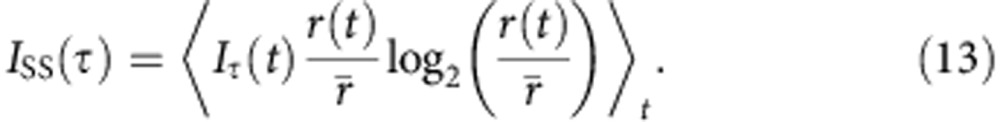




Note that while this measure of information is based on the model-predicted firing rates, and thus is expected to be an underestimate of the true single-spike information, any errors in the model-predicted response *r*(**X**) should not introduce systematic biases relative to the timing of saccades.

When comparing perisaccadic changes in *I*_SS_ across the population, we normalized *I*_SS_(*τ*) for each neuron by its average value prior to saccade onset. When comparing perisaccadic *I*_SS_(*τ*) with and without correcting for fixational eye movements (Supplementary Fig. 4), we instead normalized *I*_SS_(*τ*) by its overall average to illustrate the effects of systematic reductions in fixation error following microsaccades.

### Current source density to identify laminar boundaries

To estimate laminar boundaries we used the depth profile of stimulus-onset-triggered current sources and sinks. Current source density analysis was performed using the inverse current source density method^67^. The putative granular layer was identified by a prominent, short-latency current sink (Fig. 5c)^68^,^69^. We also used the depth profile of LFP power across a range of frequencies as a reference, utilizing the prominent peak in gamma power beginning around the upper boundary of layer IV^70^.

Each contact of the linear electrode arrays was then classified as either supragranular, granular or infragranular, based on the estimated laminar boundaries, although contacts at the boundaries were left unclassified. We then used MUA from each of the classified contacts to analyse laminar differences. Note that for analysis of laminar differences we utilized MUA, rather than only using SUs as in the rest of the analysis, to obtain sufficient statistical power, though we verified that the results were consistent using SUs.

### Timing analysis

The magnitude and timing of perisaccadic firing rate suppression (enhancement) were defined by the size and location of minima (maxima) in the saccade-triggered firing rate averages, in the range from 0 to 300 ms after saccade onset (Fig. 1c). To increase the temporal precision of our estimates, peaks were computed using spline-interpolated saccade-triggered averages. Furthermore, to avoid spurious peak detection, we computed a null distribution of peak amplitudes by randomizing saccade times relative to the spike data, and then locating peaks of the resulting event-triggered averages (using 5,000 resamples for each neuron). Only peaks whose magnitudes exceeded 95% of these ‘null peaks’ were deemed significant (computed separately for minima and maxima). For Fig. 5b, we excluded neurons where the perisaccadic firing rate enhancement occurred before suppression (8 out of 84; see Supplementary Fig. 1), though results were similar when including all neurons (correlation between firing rate suppression and stimulus response timing: 0.59; *P*=3.0 × 10^−9^).

To measure the stimulus response timing of a given neuron (Fig. 5), we used the Hilbert transform to estimate the average temporal envelope (averaged across spatial positions) of each of the neuron’s stimulus filters. We then averaged these temporal kernels across the set of excitatory subunits, weighted by the relative contribution of each subunit to the overall generating signal. This average temporal kernel was up-sampled using spline interpolation, and the location of the peak was taken as the latency of the neuron’s stimulus response.

### Additional analysis details

For analysis of triggered average firing rates, spikes were binned at 5-ms resolution and smoothed using a Gaussian kernel (sigma=7.5 ms) to estimate firing rate. Firing rate estimates were mean-normalized within each recording block of 60–90 trials. For all other analyses, spikes were binned at 10-ms resolution. For the majority of analyses (other than Fig. 7), we pooled the image background and grey background conditions, and the results in Fig. 7—where these conditions are considered separately—demonstrate the similarity of modulation in these conditions.

When evaluating the performance of models using pre-filtering versus post-filtering gain suppression (Fig. 6e), we compared the log-likelihood of the data under the two models *LL*_PRE_ and *LL*_POST_. These two models differed only by whether the perisaccadic gain kernel acted before or after a neuron’s stimulus filters (Fig. 6d), and thus had identical numbers of free parameters. We then normalized the difference (*LL*_PRE_−*LL*_POST_) by the improvement of the post-filtering model over a null model (*LL*_POST_−*LL*_NULL_), where the null model was identical, but without any perisaccadic gain modulation (having only an ‘offset’ kernel).

One-sample and paired two-sample comparisons were performed using two-sided Wilcoxon signed rank tests. Correlations and associated *P* values were computed using the nonparametric Spearman’s rank correlation (using Matlab’s corr function). Linear fits (Fig. 5b, and analysis associated with Fig. 6c) were obtained using Matlab’s robust regression routine robustfit.

For statistical analysis of the layer dependence of saccade modulation, we used a two-way ANOVA, with the putative layer and recording (*n*=9 unique laminar probe recordings) as factors (treating recording as a random effect). Statistical significance of pairwise differences between layers was then assessed using *post hoc* multiple comparisons tests (Tukey method). Boxplot whiskers depict the 10th and 90th percentiles (Fig. 5d,e).

Matlab code for estimating stimulus processing models, and performing model-based eye tracking is available for download from: http://neurotheory.umd.edu/code. Additional analysis code is available upon request.

## Additional information

**How to cite this article:** McFarland, J. M. *et al.* Saccadic modulation of stimulus processing in primary visual cortex. *Nat. Commun.* 6:8110 doi: 10.1038/ncomms9110 (2015).

## Supplementary Material

Supplementary Information Supplementary Figures 1-6
