# Supplementary material for: Saccadic modulation of stimulus processing in primary visual cortex
Source: Nat Commun. 2015 Sep 15;6:8110. doi: 10.1038/ncomms9110 (PMC4571196; doi:10.1038/ncomms9110)
Supplement: Supplementary Information — Supplementary Figures 1-6 [file ncomms9110-s1.pdf]

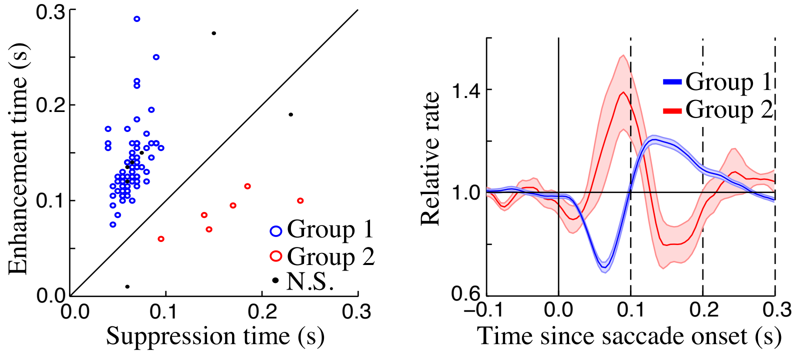

Supplementary Figure 1: A small subset of neurons showed 'opposite polarity' saccade modulation.

(*Left*) Scatterplot of the timing of perisaccadic firing rate enhancement vs. suppression. Neurons that had significant enhancement and suppression (*n*=77/84; see Methods) are shown by either blue or red circles (remaining neurons are shown as black dots). For most such neurons (*n*=71/77) the suppression preceded enhancement (blue), though for a small subset (*n*=6/77), the timing was reversed. (*Right*) Saccade-triggered average relative firing rates for the two groups of neurons. Shaded region shows the interval mean ± s.e.m.

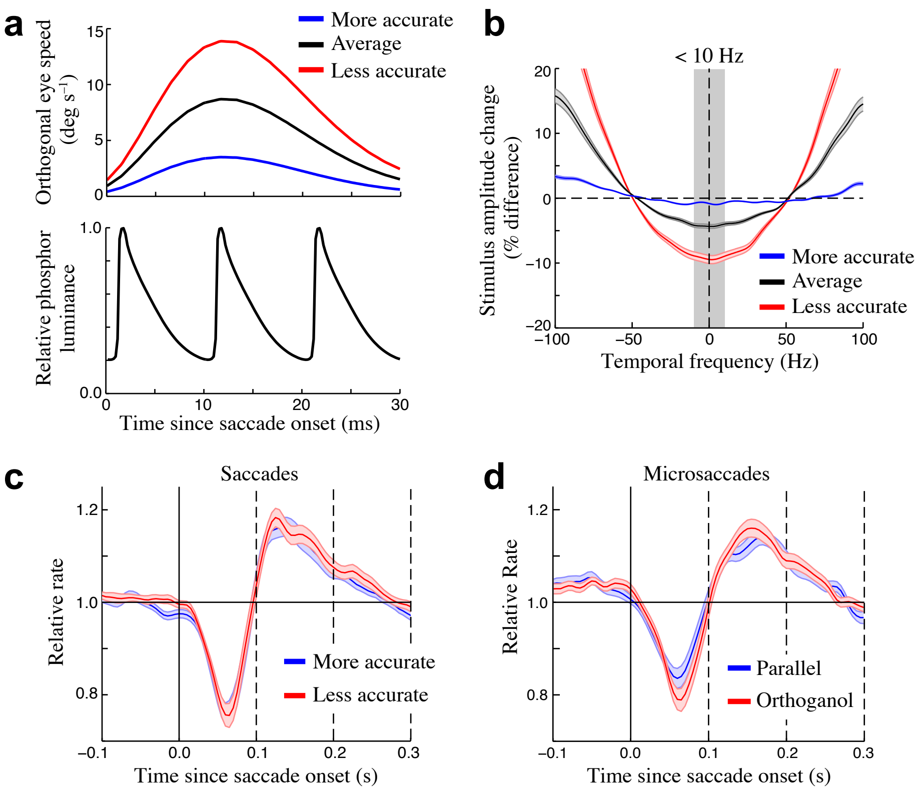

Supplementary Figure 2: Negligible effects of intrasaccadic stimulus motion.
a) We estimated the effects of saccades on the spatiotemporal statistics of the visual stimulus by reconstructing the precise image on the retina at each time point during simulated saccades. To do this we measured the phosphor luminance response of our CRT displays (*bottom*), as well as the average perisaccadic eye velocity profile in the dimension orthogonal to the bar stimuli (*top*; Methods). We then computed the power spectra of the stimulus on the retina during saccades as well as without eye movement. We also performed the analysis separately using the velocity profile of the 'most accurate' (blue) and 'least accurate' (red) set of saccades (using a median split). **b**) At the preferred spatial frequencies of the recorded neurons, the temporal frequency content of the stimulus during saccades was only slightly changed compared to during static fixation. In particular, at relevant temporal frequencies (the central shaded gray region indicates frequencies < 10 Hz), the average saccade produced < 5% reduction in the stimulus amplitude. For the most accurate saccades these reductions were < 1%, while for the least accurate saccades the changes were still < 10%. Relative changes in temporal frequency spectra were computed at the preferred spatial frequencies of each SU used in our analysis (n=84), and the shaded regions show the region mean $\pm$ s.e.m. across SUs. **c**) To verify that inaccuracies in saccades, and hence any orthogonal displacement of the stimulus pattern during saccades, did not contribute to the observed perisaccadic modulation we computed saccade-triggered average firing rates separately for the most and least accurate sets of saccades. The firing rate modulation was nearly identical for both groups of saccades, showing that saccade accuracy was not a factor in the observed modulation. **d**) We performed a similar analysis to determine whether motion of the retinal stimulus during microsaccades contributed to the observed modulation of V1 responses. In this case we separated microsaccades into those that were more parallel (blue) vs. more orthogonal (red) to the bar stimuli (see Methods). Orthogonal microsaccades produced only slightly stronger suppression (median 1.12-fold; p=2.2x10^{-5}; n=88), and enhancement (1.09-fold; p=0.048) of single unit firing rates compared with parallel microsaccades, again showing that intrasaccadic stimulus motion was not a significant factor in the observed effects.

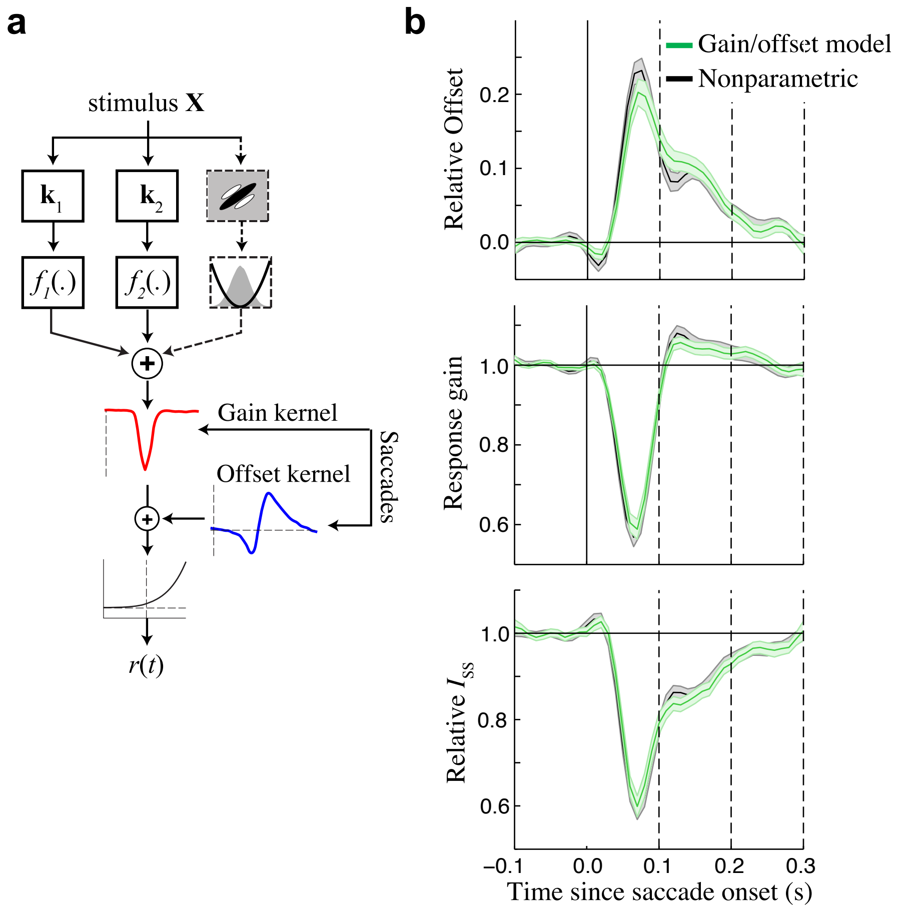

Supplementary Figure 3: Simple 'gain/offset' model captures perisaccadic changes in response gain, offset, and stimulus selectivity.

a) Schematic showing an LNLN model with a multiplicative perisaccadic gain kernel (red) applied to the summed output of the subunits, as well as an additive perisaccadic offset kernel (blue). **b**) From top to bottom, average response offset, gain, and I_{SS} computed using the gain/offset model (green) compared with the nonparametric model (black). The gain/offset model is able to capture a very similar description of saccade modulation as the nonparametric methods. We also verified that the model with both gain and offset kernels performed significantly better than either a gain-only (p=9.3x10^{-13}; n=84) or an offset-only (p=2.5x10^{-8}) model (using a random subset of 20% of trials to evaluate the cross-validated LL).

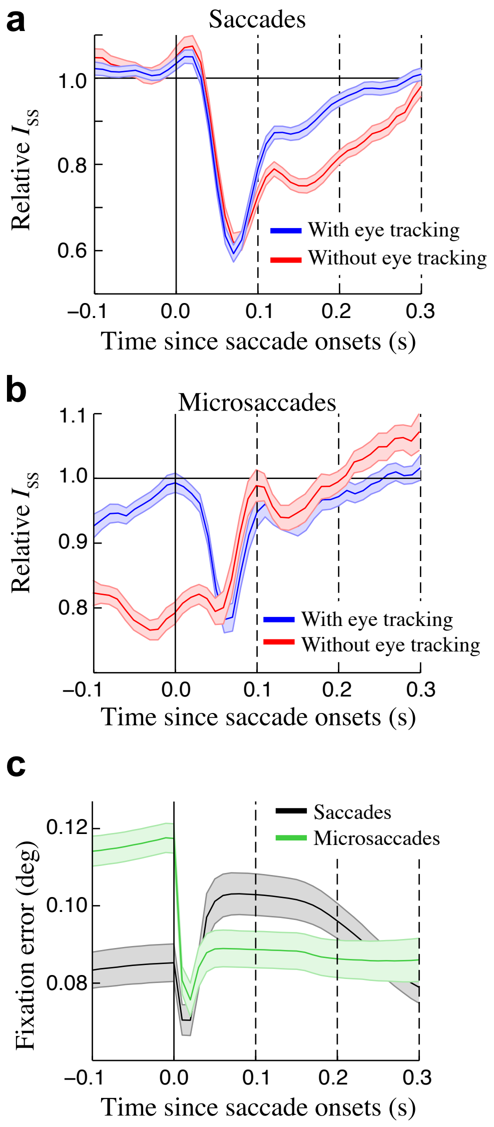

Supplementary Figure 4: Dependence of results on eye-tracking.
a) Relative changes in single-spike information (I_{SS}) following saccades were qualitatively similar with (blue) and without (red) correcting for inferred eye position. This is despite the fact that there was a nearly two-fold reduction in overall I_{SS} when corrections for eye position were not incorporated (median ratio of I_{SS} without to with corrections: 0.58; n=84). **b**) For microsaccade modulation, incorporating corrections for eye position was more important. Without eye tracking, the relative I_{SS} appeared to increase suddenly following microsaccades. **c**) This apparent increase in I_{SS} following microsaccades when assuming perfect fixation is due to the fact that microsaccades tend to be corrective (i.e., they reduce fixation error). Average fixation error following saccades (n=17 recordings; black) and microsaccades (red). Microsaccades produced a sharp reduction in fixation error, while saccades were followed by more modest increases in fixation error.

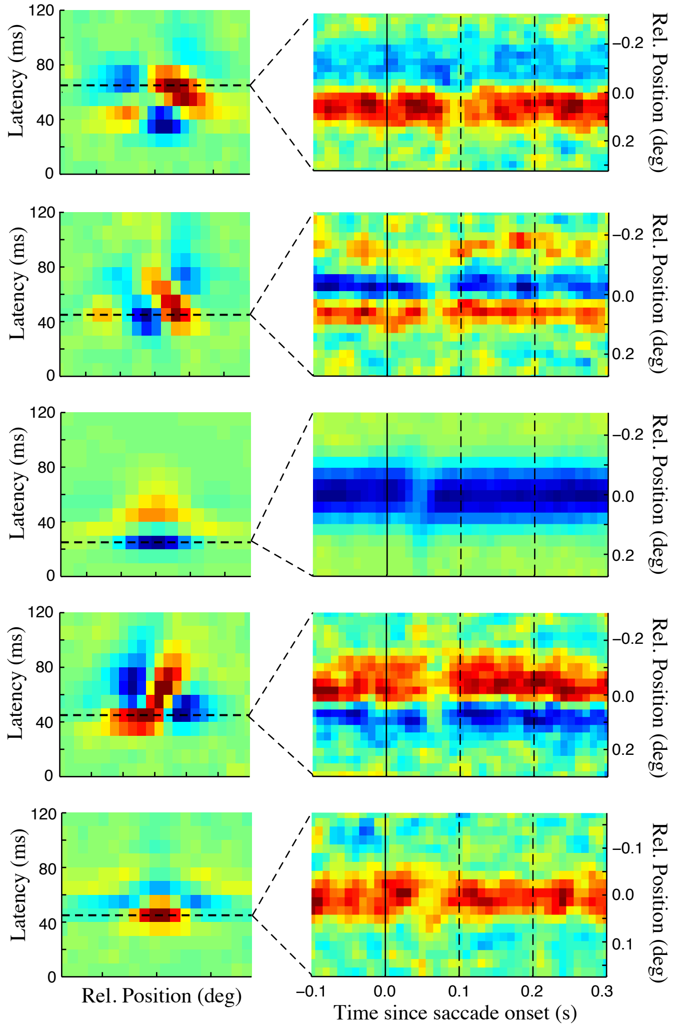

Supplementary Figure 5: Example saccade-conditional spike-triggered averages.
(*Left*) Spike triggered average (STA) stimuli computed for five example neurons. The optimal latency (latency with maximal spatial variance in the STA) is indicated by the dashed horizontal lines. (*Right*) For each neuron, the spatial profile of the STA (at the optimal latency) was computed at each time relative to saccade onset. Saccade-conditional STAs were smoothed slightly in space with a Gaussian kernel. While clear perisaccadic changes in the STA were difficult to resolve (being limited largely by the number of available saccades), those cases with clear structure showed only a brief decrease in STA amplitude following saccade.

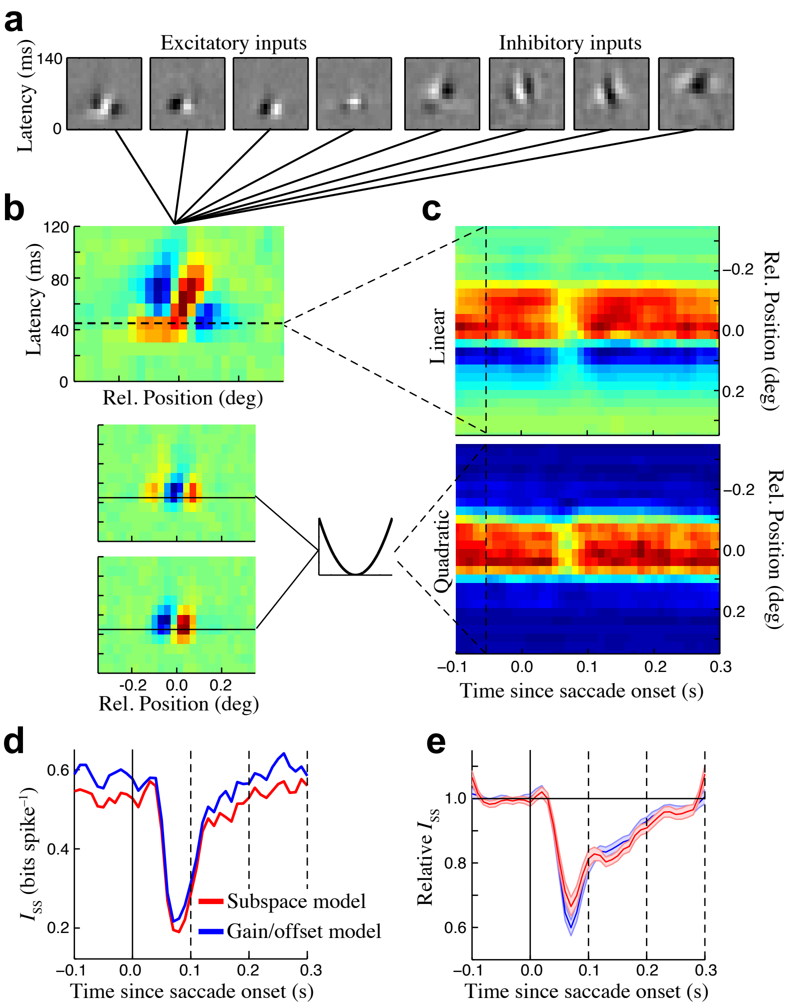

Supplementary Figure 6: No evidence for more general forms of saccade modulation. (**a-c**) We estimated models where a neuron's stimulus filters were allowed to vary as a function of time relative to saccade onset. To maintain tractability, we assumed that the neuron's filters could be represented as linear combinations of a set of basis vectors, given by the set of stimulus filters recovered for that neuron. **a)** The stimulus filters for an example neuron. **b**) Each of the neuron's stimulus filters in the 'subspace' model is then represented as a separate linear combination of these basis filters at each time relative to saccade onset. (*Top*) The linear filter at an example time relative to saccade onset (indicated by the dashed vertical line in c), along with the corresponding quadratic filters (*Bottom*) **c**) The perisaccadic spatial tuning (at the optimal latency) for the same example neuron. Both the linear component of stimulus tuning (*top*), and the quadratic component (*bottom*; vector magnitude of the squared filters) showed brief suppression following the saccades, but no other clear structure. **d**) Perisaccadic changes in stimulus information (I_{SS}) captured by the subspace model (red) were similar to those described by the simple gain/offset model (blue). Note the subspace model captures slightly less stimulus information overall, as it uses fewer subunits to maintain tractability. **e**) Across the population, the relative changes in I_{SS} were similar for the subspace model (red) and the gain/offset model (blue).
